# First crystal structure of the DUF2436 domain of virulence proteins from *Porphyromonas gingivalis*

**DOI:** 10.1107/S2053230X24008185

**Published:** 2024-09-26

**Authors:** Bogeun Kim, Jisub Hwang, Sehyeok Im, Hackwon Do, Youn-Soo Shim, Jun Hyuck Lee

**Affiliations:** a Division of Life Sciences, Korea Polar Research Institute, Incheon21990, Republic of Korea; b Department of Polar Sciences University of Science and Technology Incheon21990 Republic of Korea; c Department of Dental Hygiene, Sunmoon University, Asan31460, Republic of Korea; MAX IV Laboratory, Sweden

**Keywords:** DUF2436 domain, oral pathogens, *Porphyromonas gingivalis*, X-ray crystallography, cysteine proteases

## Abstract

*Porphyromonas gingivalis* is a major pathogenic oral bacterium that is responsible for periodontal disease. It is linked to chronic periodontitis, gingivitis and aggressive periodontitis. *P. gingivalis* exerts its pathogenic effects through mechanisms such as immune evasion and tissue destruction, primarily by secreting various factors, including cysteine proteases such as gingipain K (Kgp), gingipain R (RgpA and RgpB) and PrtH (UniProtKB ID P46071). Virulence proteins comprise multiple domains, including the pro-peptide region, catalytic domain, K domain, R domain and DUF2436 domain. While there is a growing database of knowledge on virulence proteins and domains, there was no prior evidence or information regarding the structure and biological function of the well conserved DUF2436 domain. In this study, the DUF2436 domain of PrtH from *P. gingivalis* (*Pg*DUF2436) was determined at 2.21 Å resolution, revealing a noncanonical β-jelly-roll sandwich topology with two antiparallel β-sheets and one short α-helix. Although the structure of *Pg*DUF2436 was determined by the molecular-replacement method using an *AlphaFold* model structure as a template, there were significant differences in the positions of β1 between the *AlphaFold* model and the experimentally determined *Pg*DUF2436 structure. The *Basic Local Alignment Search Tool* sequence-similarity search program showed no sequentially similar proteins in the Protein Data Bank. However, *DaliLite* search results using structure-based alignment revealed that the *Pg*DUF2436 structure has structural similarity *Z*-scores of 5.9–5.4 with the C-terminal domain of AlgF, the D4 domain of cytolysin, IglE and the extracellular domain structure of PepT2. This study has elucidated the structure of the DUF2436 domain for the first time and a comparative analysis with similar structures has been performed.

## Abstract 2

The DUF2436 domain of unknown function is highly conserved in virulence proteins from the pathogenic oral bacterium *P. gingivalis*. Theis domain exhibits a noncanonical β-jelly-roll sandwich topology with a previously unobserved fold consisting of two antiparallel β-sheets and one short α-helix.

## 1. Introduction

*Porphyromonas gingivalis* is a Gram-negative, anaerobic oral pathogenic bacterium that is involved in the early onset and progression of periodontitis (Brown *et al.*, 1996 ▸). The symptoms of periodontal disease caused by *P. gingivalis* include red, swollen and bleeding gums, receding gums, persistent bad breath, painful chewing, loose teeth, pus between the teeth and gums, and new spaces between teeth (Armitage, 2004 ▸; Pihlstrom *et al.*, 2005 ▸). *P. gingivalis* induces an imbalance in the oral microbiome, allowing increased numbers of perio­dontal pathogenic bacteria and fungi to induce chronic inflammation. *P. gingivalis* can produce various virulence factors, including lipopolysaccharide, fimbriae/pili, collagenase, lectins, capsules, superoxide dismutase and various proteases such as gingipains, that evade the host immune defense system and destroy host periodontal tissues (Jia *et al.*, 2019 ▸; Ebersole *et al.*, 2017 ▸).

Gingipains are trypsin-like cysteine proteases that include arginine-specific proteinases (RgpA and RgpB) and lysine-specific proteinases (Kgps) (Nakayama *et al.*, 1996 ▸; Okamoto *et al.*, 1996 ▸; Potempa *et al.*, 2003 ▸). Gingipain is a multi-domain protease with membrane-bound and extracellular forms. The proteolytic enzymes are initially expressed as large pre-pro-proteins that undergo complex and poorly elucidated processes of maturation, activation and secretion. *P. gingivalis* secretes gingipains to degrade host cytokines, thereby evading the immune response. The bacteria also break down host hemoglobin and then utilize heme as an iron source for their growth and survival (Hajishengallis *et al.*, 2020 ▸). RgpB consists of one larger subunit representing the catalytic domain of the enzyme followed by a short C-terminal region (Seers *et al.*, 2006 ▸). In contrast, Kgp and RgpA consist of multiple domains and subunits; in particular, each has a pro-peptide region, a catalytic domain, two or three K domains and one or two domains of unknown function (DUFs) known as DUF2436 (Dashper *et al.*, 2017 ▸). Currently, no structural information is available for full-length gingipain proteins, only for certain domains analyzed by X-ray crystallography, such as the catalytic and IgSF (immunoglobulin-superfamily) domains of RgpB (Eichinger *et al.*, 1999 ▸), the C-terminal domain of RgpB (Seers *et al.*, 2006 ▸), the catalytic and IgSF domains of Kgp (de Diego *et al.*, 2014 ▸), the K1 domain of Kgp (Ganuelas *et al.*, 2013 ▸), the K2 domain of Kgp (Li *et al.*, 2010 ▸), the K3 domain of Kgp (Li *et al.*, 2011 ▸) and the N-terminal pro-domain of Kgp (Pomowski *et al.*, 2017 ▸). Although the structure and function of the catalytic domain and some K domains of gingipains are known, the structure and function of DUF2436 have not been characterized (Potempa *et al.*, 2003 ▸; Li & Collyer, 2011 ▸). However, DUF2436 domains might be crucial for enzymatic activity since they exhibit conserved amino-acid sequences (over 62% sequence similarity) within gingipains.

In 1994, a new putative protease gene (*prtH*) from *P. gingivalis* was identified and characterized (Fletcher *et al.*, 1994 ▸). Although its detailed function has not been elucidated, PrtH contains 989 amino-acid residues and multiple domains, including a cleaved adhesion domain, small Ig-like fold domains and a DUF2436 domain (Li *et al.*, 2010 ▸).

We determined the crystal structure of the DUF2436 domain of PrtH at a resolution of 2.21 Å for the first time. This domain is encoded by a protease involved in the periodontitis-inducing mechanisms of *P. gingivalis*. We recombinantly expressed the DUF2436 domain (residues Ser361–Cys579) of PrtH (UniProtKB ID P46071) from *P. gingivalis* in *Escherichia coli*. Results from a *Basic Local Alignment Search Tool* (*BLAST*) search revealed that no homologous proteins with a similar structure to *Pg*DUF2436 have previously been reported (Johnson *et al.*, 2008 ▸). Therefore, this study is expected to provide valuable insights for elucidating and understanding the structure of DUF2436 and similar domains.

## 2. Materials and methods

### 2.1. Expression and purification of *Pg*DUF2436

The information regarding the *P. gingivalis* genome (strain ATCC BAA-308/W83) is already registered in the NCBI database (Nelson *et al.*, 2003 ▸). The gene encoding *Pg*DUF2436 from PrtH (UniProtKB ID P46071, amino-acid residues Ser361–Cys579) was selected to study the crystal structure since it is conserved in other gingipains (Bateman *et al.*, 2023 ▸). The gene was synthesized and cloned into the pET-28a vector using the NdeI and XhoI restriction enzymes. The cloned sequence was confirmed by sequencing with T7 promoter and T7 terminator primers (Table 1 ▸).

The recombinant plasmid was transformed into *E. coli* BL21 (λDE3) competent cells. Transformed *E. coli* cells were cultured in 1 l Luria–Bertani medium with 50 µg ml^−1^ kanamycin at 37°C and incubated at 150 rev min^−1^. When the optical density of the cells at 600 nm reached 0.4, 1 m*M* isopropyl β-d-1-thiogalactopyranoside was added to induce overexpression. The culture was then incubated at 25°C and 120 rev min^−1^ for 16 h. The cells were harvested by centrifugation at 6000 rev min^−1^ for 20 min at 4°C. The collected cells were resuspended in 50 ml buffer *A *(20 m*M* Tris–HCl pH 8.0, 200 m*M* NaCl) and then disrupted using sonication (Vibra-Cell, Sonics & Materials, Danbury, Connecticut, USA) for 30 min (32% amplitude, 2 s/5 s pulse and rest at 4°C). The soluble protein fraction was separated via ultracentrifugation at 4°C and 13 000 rev min^−1^ for 50 min.

For His-tag affinity purification, an Ni–NTA (nickel-charged affinity resin) column was pre-washed with five column volumes of distilled water and equilibrated with buffer *B* (20 m*M* Tris–HCl pH 8.0, 200 m*M* NaCl, 30 m*M* imidazole). The supernatant was loaded onto the Ni–NTA column to bind *Pg*DUF2436 to the nickel-charged resin. Non-targeted proteins and debris were washed out with five column volumes of buffer *B*, and the bound *Pg*DUF2436 was eluted using two column volumes of buffer *C* (20 m*M* Tris–HCl pH 8.0, 200 m*M* NaCl, 300 m*M* imidazole). To prevent interference by imidazole during the thrombin reaction, the collected *Pg*DUF2436 was processed via buffer exchange from buffer *C* to buffer *A* using a 10 kDa cutoff Amicon Ultra-15 centrifugal filter unit tube (Merck, Darmstadt, Germany). To cleave the 6×His tag, 80 units of thrombin were added to the *Pg*DUF2436-containing buffer *A* and reacted for 72 h. For further purification and to remove thrombin, size-exclusion chromatography was performed with buffer *A* on a HiLoad 16/600 Superdex 200 pg column (Cytiva, Marlborough, Massachusetts, USA). The purified *Pg*DUF2436 protein was concentrated to 120 mg ml^−1^ using a 10 kDa cutoff Amicon Ultra-15 centrifugal filter unit. The final purified *Pg*DUF2436 protein was evaluated by 12% sodium dodecyl sulfate–polyacrylamide gel electrophoresis. The concentrated *Pg*DUF2436 protein was stored at −80°C.

### 2.2. Protein crystallization and X-ray diffraction data collection

The frozen *Pg*DUF2436 was thawed on ice until it was stabilized at 4°C. The aggregated pellet was removed and only soluble *Pg*DUF2436 was prepared for crystallization at a concentration of 90 mg ml^−1^. *Pg*DUF2436 was crystallized by the sitting-drop vapor-diffusion method using a crystallization screening solution kit (Anatrace, Maumee, Ohio, USA). After mixing the crystallization solution and *Pg*DUF2436 in a 1:1 ratio (300:300 nl), crystallization was conducted using an MRC 2-lens crystallization plate (SWISSCI, High Wycombe, UK) with a Mosquito LCP crystallization robot (SPT Labtech, Hertfordshire, UK). After seven days, *Pg*DUF2436 crystals were observed under a condition consisting of 0.2 *M* sodium chloride, 0.1 *M* Tris–HCl pH 7.0, 1 *M* sodium citrate (Table 2 ▸). The crystals grew to a maximum length of approximately 200 µm. A single *Pg*DUF2436 crystal was soaked briefly in a cryoprotectant solution consisting of glycerol added to the crystallization solution, resulting in a final glycerol concentration of 20%. Using this crystal, 360 X-ray diffraction images (each containing 1° of oscillation) were collected on the 7A-SB I beamline at Pohang Accelerator Laboratory (PAL), Pohang, Republic of Korea.

### 2.3. Structure determination and refinement

X-ray diffraction data were processed using *XDS* (Kabsch, 2010 ▸). *POINTLESS* and *AIMLESS* were employed to determine the point and space groups (Evans, 2011 ▸; Agirre *et al.*, 2023 ▸). The initial search model structure was built using an *AlphaFold* model as a molecular-replacement model (Jumper *et al.*, 2021 ▸). The asymmetric unit content was defined as a monomer using the Matthews coefficient value (Matthews, 1968 ▸; Vagin & Teplyakov, 2010 ▸; Jumper *et al.*, 2021 ▸). Subsequent automatic structure refinement was performed using *REFMAC*5 and *Phenix*, followed by manual model building and correction using *Coot* (Liebschner *et al.*, 2019 ▸; Emsley *et al.*, 2010 ▸; Murshudov *et al.*, 2011 ▸). The refined structure was validated using *MolProbity* (Williams *et al.*, 2018 ▸) and visualized using *PyMOL* (DeLano, 2002 ▸). The final *Pg*DUF2436 structure was deposited in the Protein Data Bank (PDB) with the accession code 9isp. A summary of the data-collection and refinement statistics is presented in Table 3 ▸.

## 3. Results and discussion

### 3.1. First structure of *Pg*DUF2436

**Figure 1:**
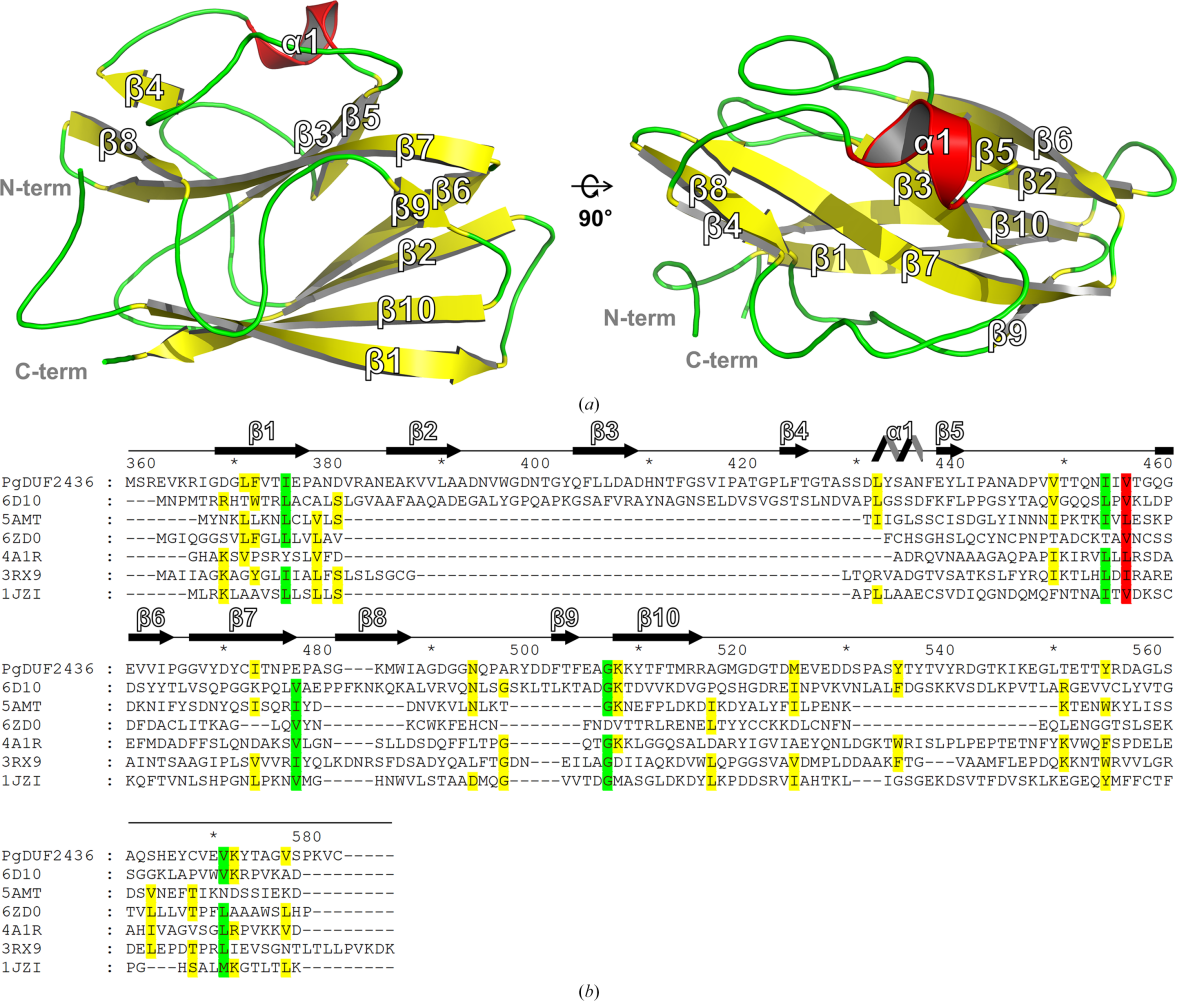
Overall monomeric structure of *Pg*DUF2436 and multiple sequence alignment of *Pg*DUF2436. (*a*) Overall structure of *Pg*DUF2436, shown as a cartoon model using *PyMOL* (DeLano, 2002 ▸). The N- and C-termini are labeled, and the figure on the right shows the *Pg*DUF2436 structure rotated 90° around the *x* axis. (*b*) Multiple sequence alignment of *Pg*DUF2436 (UniProtKB ID P46071, PDB entry 9isp), the C-terminal domain of AlgF from *Pseudomonas aeruginosa* (UniProtKB ID Q06062, PDB entry 6d10), intracellular growth locus E protein (IglE) from *Francisella tularensis* (UniProtKB ID A0Q7H2, PDB entry 5amt), the D4 domain of cholesterol-dependent cytolysin from *Streptococcus intermedius* (UniProtKB ID Q9LCB8, PDB entry 6zd0), *Serratia marcescens* Lip, a membrane-bound component of the type VI secretion system (UniProtKB ID G5EA77, PDB entry 4a1r; Rao *et al.*, 2011 ▸), TssJ from the bacterial type VI secretion system from *E. coli* (UniProtKB ID B7LFS8, PDB enty 3rx9; Felisberto-Rodrigues *et al.*, 2011 ▸) and electron-transport protein from *Pseudomonas aeruginosa* (UniProtKB ID P00282, PDB code 1jzi; Crane *et al.*, 2001 ▸). The alignment sequences have been chosen from the top hit results of a *DaliLite* server search (PDB entries 6d10, 5amt and 6zd0) and the *TM-align* top hit results of a *COFACTOR* server search (PDB entries 4a1r, 3rx9 and 1jzi; Zhang & Skolnick, 2005 ▸; Roy *et al.*, 2012 ▸; Zhang *et al.*, 2017 ▸). The alignment was performed by *ClustalX* (Larkin *et al.*, 2007 ▸) and visualized using *GeneDoc* (Nicholas *et al.*, 1997 ▸). Secondary-structural elements in the crystal structure of *Pg*DUF2436 are represented above the sequence.

Purified recombinant *Pg*DUF2436 was successfully crystallized by the sitting-drop vapor-diffusion method at 293 K. The crystal belonged to space group *P*3_2_21 and contained a monomer of *Pg*DUF2436 in the asymmetric unit. The crystal structure of *Pg*DUF2436 was determined at 2.21 Å resolution (Supplementary Fig. S1). Structural refinement resulted in *R*-factor and *R*_free_ values of 23.7% and 25.8%, respectively. Met516–Cys579 were not modeled in this structure because of very weak electron density in this region. The refined structure of *Pg*DUF2436 consists of ten β-strands and one short α-helix (Fig. 1 ▸).

Fig. 1 ▸(*b*) shows the multiple sequence alignment results, which reveal that the amino-acid sequence of the *Pg*DUF2436 domain differs significantly from those of proteins with known structures. This difference persists despite the identification of structural analogs by the *DaliLite* (Holm, 2022 ▸) and *COFACTOR* servers (Roy *et al.*, 2012 ▸; Zhang *et al.*, 2017 ▸), which are designed to detect structural similarities and analogs using methods such as *TM-Align* (Supplementary Table S1; Zhang & Skolnick, 2005 ▸).

**Figure 2:**
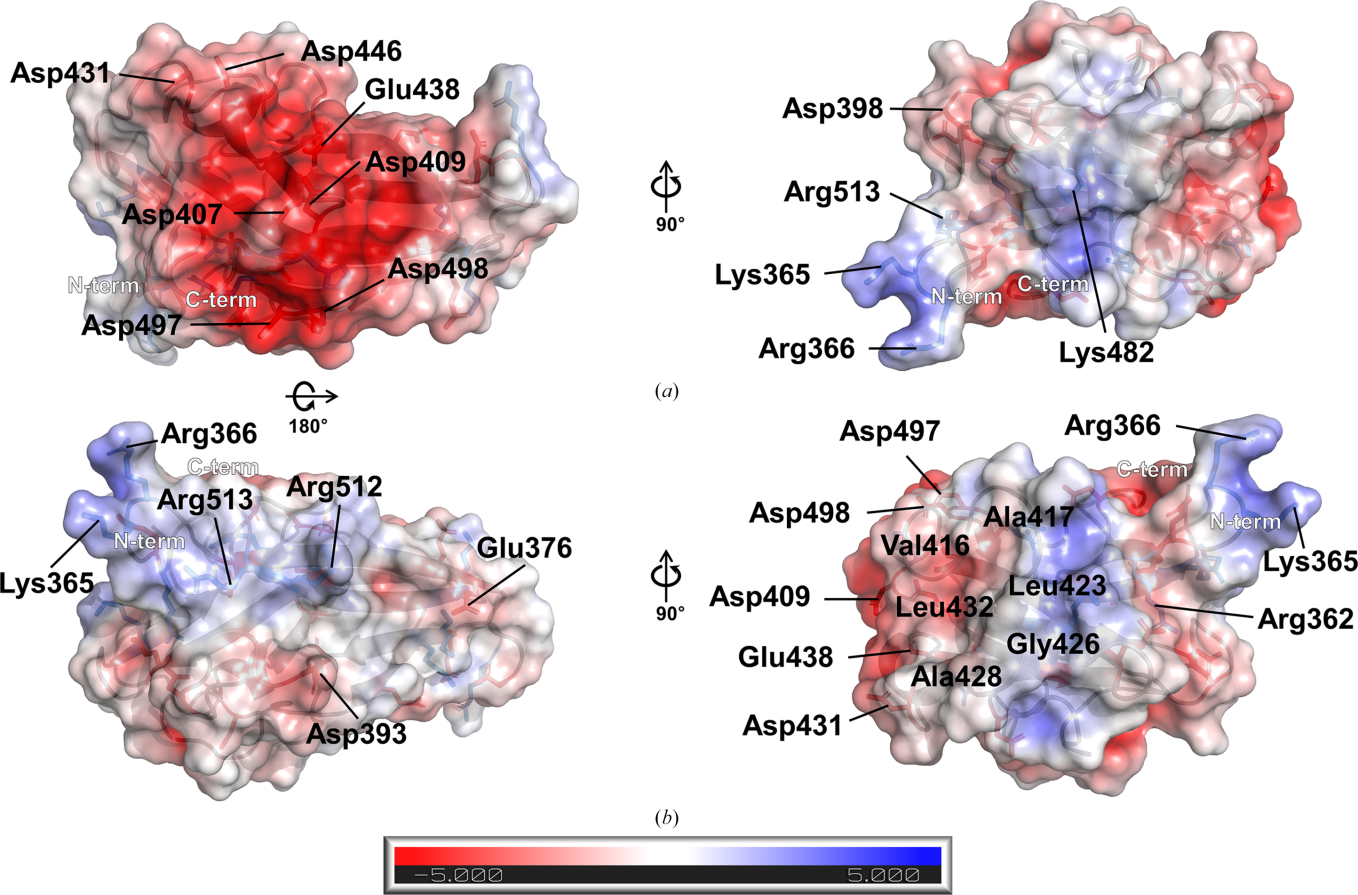
(*a*) Electrostatic surface-charge representation of the *Pg*DUF2436 domain structure. (*b*) The structure in (*a*) rotated 180°. The electrostatic surface potential was calculated using *APBS* and is colored according to calculated charge from red (−5 *kT*/e) to blue (+5 *kT*/e).

The calculated molecular weight of *Pg*DUF2436 was about 26 kDa based on the amino-acid sequence. The size-exclusion chromatography result indicated that *Pg*DUF2436 exists as a monomer in solution (Supplementary Fig. S2). Analysis of the electrostatic surface charge of the *Pg*DUF2436 structure reveals the presence and location of negatively charged patches (Fig. 2 ▸). The negatively charged patch on β3, β5, β8 and β9 is formed by Asp407, Asp409, Asp431, Asp446, Glu438, Asp497 and Asp498.

**Figure 3:**
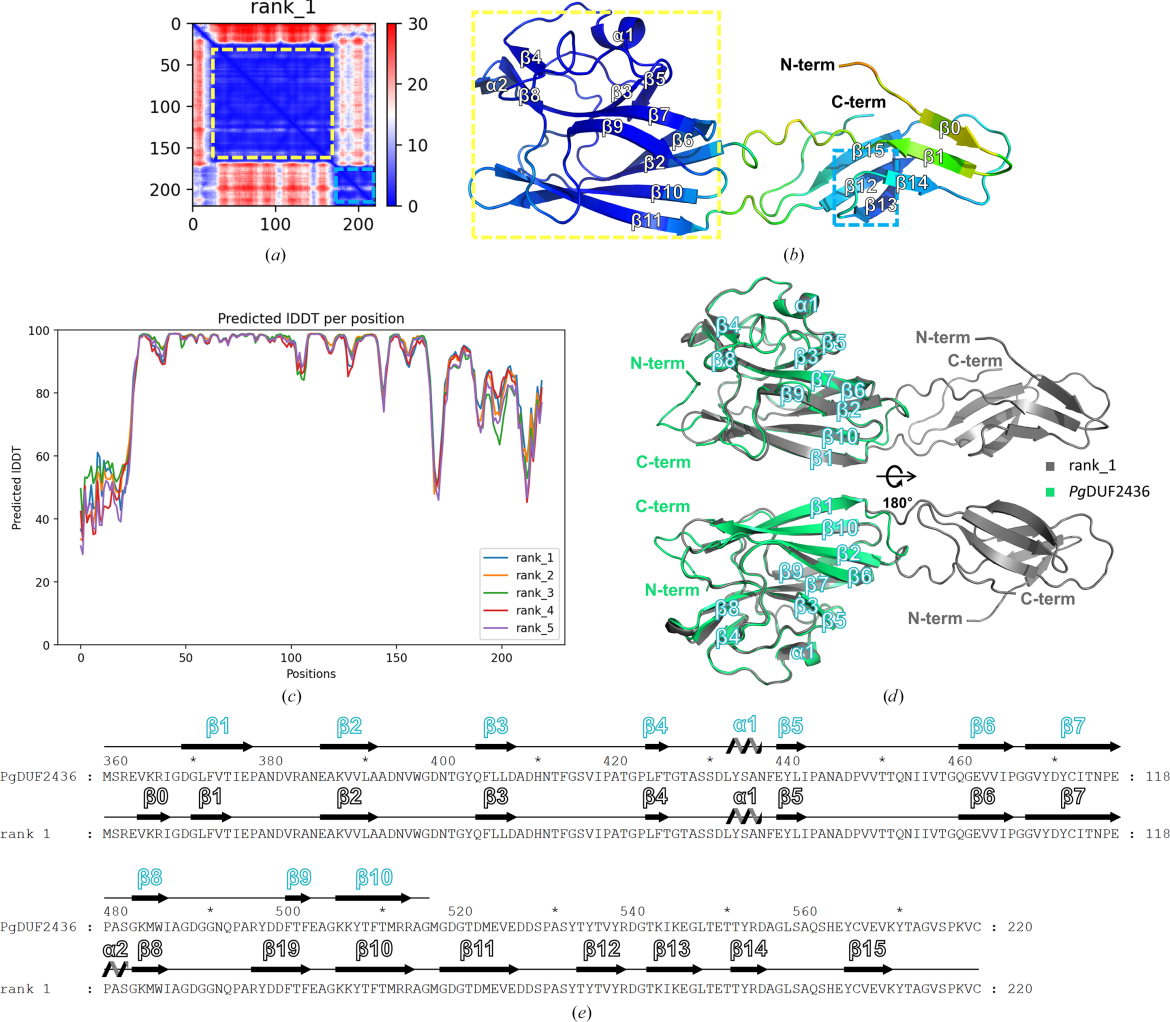
Structural comparison of the *AlphaFold* model and crystal structure of *Pg*DUF2436. (*a*) The predicted aligned error (PAE) plots are shown as a heatmap image with color-coded high confidence (blue) to low confidence (red), where the *x* and *y* axes correspond to residues. (*b*) The top-ranked *AlphaFold* model rank_1 is shown as a cartoon, colored by the predicted local distance difference test (plDDT) score, ranging from blue (over 90) to red (less than 50). The dashed yellow and cyan lines indicate the subdomain boundaries in the *AlphaFold* model. The yellow dotted box highlights the region from Asn384 to Glu525, while the cyan-colored dotted box indicates the region from Tyr535 to Cys579. (*c*) The plDDT score is plotted per residue for the top five ranked *AlphaFold* models. (*d*) Superposition of the experimentally determined *Pg*DUF2436 domain structure (green, secondary-structure labeling in white text with a green border) with the *AlphaFold* prediction model (gray). (*e*) Secondary-structure alignment of *Pg*DUF2436 and rank_1. The largest difference in the comparison between the crystal structure and the *AlphaFold* model (rank_1) of *Pg*DUF2436 is the determination of domain boundaries. The rank_1 structure predicted that the *Pg*DUF2436 domain consists of β0–β11 (residues Glu385–Asp526), but the actual crystal structure shows that the *Pg*DUF2436 domain is composed of β1–β10 (residues Asp369–Arg513). The region corresponding to residues Met516–Cys579 (β11–β15) was absent from the actual *Pg*DUF2436 crystal structure because of very weak electron density.

Fig. 3 ▸ shows the top five structural models of *Pg*DUF2436 generated by *AlphaFold*. In a predicted aligned error (PAE) plot analysis, we observed a large blue square around residues Asn384–Glu525. This implies that this region has an ordered predicted model with high confidence and low error. We used the *AlphaFold* model structure as an MR template model to solve the structure of *Pg*DUF2436. However, there are significant differences between the *AlphaFold* model and the actual *Pg*DUF2436 structure. The model predicts that β0 and β1 are positioned externally, unlike in the actual structure (Fig. 3 ▸). In the structure of *Pg*DUF2436, β1 interacts with β10, forming a β-sheet. In conclusion, *AlphaFold* predicted some parts of the *Pg*DUF2436 domain structure relatively accurately, but incorrectly predicted the position of β1, leading to an overall incorrect topology. This result suggests that the *Pg*DUF2436 domain possesses a unique fold that cannot be accurately predicted using current prediction programs.

**Figure 4:**
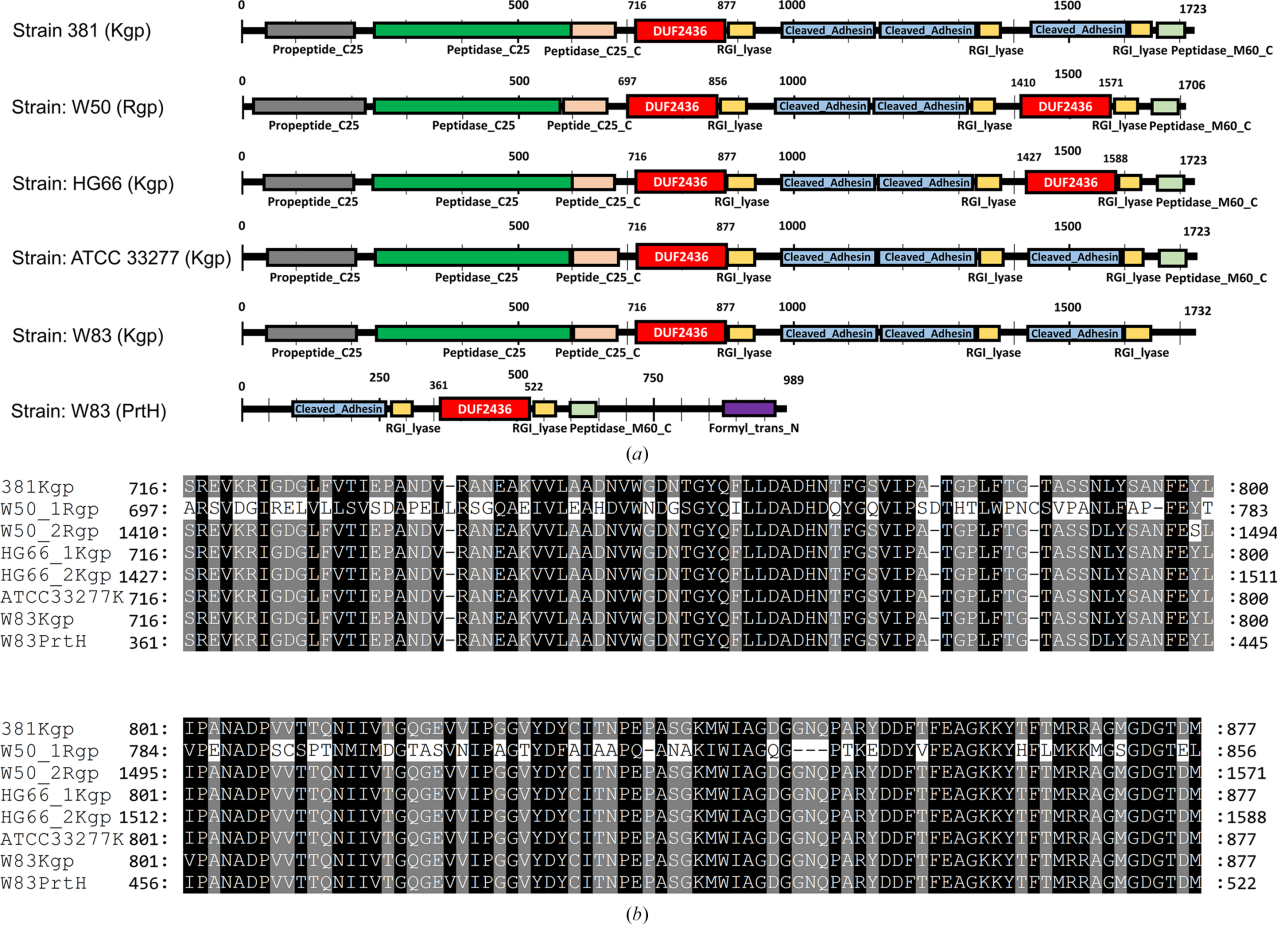
The highly conserved DUF2436 domain of PrtH and gingipains (Kgp and Rgp). (*a*) Domain organization of *P. gingivalis* strain 381 Kgp (UniProtKB ID P72194), *P. gingivalis* strain W50 Rgp (UniProtKB ID Q51839), *P. gingivalis* strain HG66 Kgp (UniProtKB ID P72197), *P. gingivalis* strain ATCC 33277 Kgp (UniProtKB ID B2RLK2), *P. gingivalis* strain W83 Kgp (UniProtKB ID Q51817) and *P. gingivalis* strain W83 PrtH (UniProtKB ID P46071). The DUF2436 domain is highlighted in a red box with white text. (*b*) A multiple sequence alignment of DUF2436 domains in PrtH and gingipains (Kgp and Rgp) reveals that the DUF2436 domain sequence is highly conserved across several strains. Notably, the first DUF2436 domain of W50 Rgp shows significant divergence from the conserved sequence (white background). The sequence alignments and analyses were performed using *ClustalX* (Larkin *et al.*, 2007 ▸) and were visualized using *GeneDoc* (Nicholas *et al.*, 1997 ▸).

The multiple amino-acid sequence-alignment results using *Pg*DUF2436 and other DUF2436 domain sequences found in gingipains showed that the DUF2436 domain is highly conserved in the virulence proteins (gingipains and PrtH) of *P. gingivalis*, with a sequence-similarity level of greater than 95%, except for the first DUF2436 domain of Rgp from *P. gingivalis* strain W50. We performed multiple sequence alignments of *Pg*DUF2436 and DUF2436 domains located in five different strains of *P. gingivalis* (UniProtKB IDs P72194, Q51839, P72197, B2RLK2, Q51817 and P46071). Notably, Rgp from *P. gingivalis* strain W50 (UniProtKB ID Q51839) and Kgp from *P. gingivalis* strain HG66 (UniProtKB ID P72197) contained two copies of the DUF2436 domain (Fig. 4 ▸).

Notably, the first DUF2436 domain (Arg697–Leu856) of Rgp from *P. gingivalis* strain W50 differed significantly from the other DUF2436 domains. From the results of this multiple sequence alignment, it was possible to distinguish between the relatively conserved regions (Leu742–His747, Val753–Pro755, Asn782–Pro785, Phe835–Tyr841 and Gly851–Thr854) in the first DUF2436 domain of Rgp from *P. gingivalis* strain W50 and the part with variation in amino-acid sequence (Leu709–Ile725, His759–Pro774, Ser786–Asn801, Phe809–Ile820 and His842–Ser850). Notably, Kgp from strain HG66 contains an additional DUF2436 domain compared with Kgp from strain W83. The reasons for the presence of multiple DUF2436 domains in a single protein and the variation in the number of DUF2436 domains across different strains warrant further investigation.

Next, a structural homolog search was performed with the *DaliLite* server and the PDB using *Pg*DUF2436 as the query (Table 4 ▸; Holm, 2022 ▸). The *DaliLite* server is a bioinformatics tool for comparing protein structures. It provides researchers with a valuable resource for exploring relationships and functional implications based on structural similarity rather than sequence alone. The results include a list of proteins with similar structures ranked by a structural similarity score, known as the *DaliZ*-score. A higher *DaliZ*-score signifies more significant structural similarity between proteins. Generally, a *Z*-score exceeding 2.0 is considered to be statistically significant, suggesting that the observed structural alignment is unlikely to be due to random chance (Holm, 2020 ▸). The results showed the highest structural similarity to be to AlgF (PDB entry 6d10; an adaptor protein) from *Pseudomonas aeruginosa*, with a *Z*-score of 5.9 (Holm *et al.*, 2008 ▸; Low *et al.*, 2023 ▸). The sequence identity between *Pg*DUF2436 and AlgF is only 9%. To compare the structures of *Pg*DUF2436 and AlgF, structural superposition and alignment were conducted using *PyMOL* and *SPDB viewer* (Kaplan & Littlejohn, 2001 ▸; DeLano, 2002 ▸). The structures of these two proteins did not generally overlap. The results showed low structural similarity and different conformations with large displacements. A *BLASTp* search was also performed using the PDB with the *Pg*DUF2436 amino-acid sequence as a query to identify similar structural homologs. The results showed that there are no known structural homologs of *Pg*DUF2436. Collectively, these results demonstrate that *Pg*DUF2436 exhibits a novel structure with a modified β-jelly-roll sandwich fold topology.

### 3.2. Structural comparison of *Pg*DUF2436 with other protein structures

**Figure 5:**
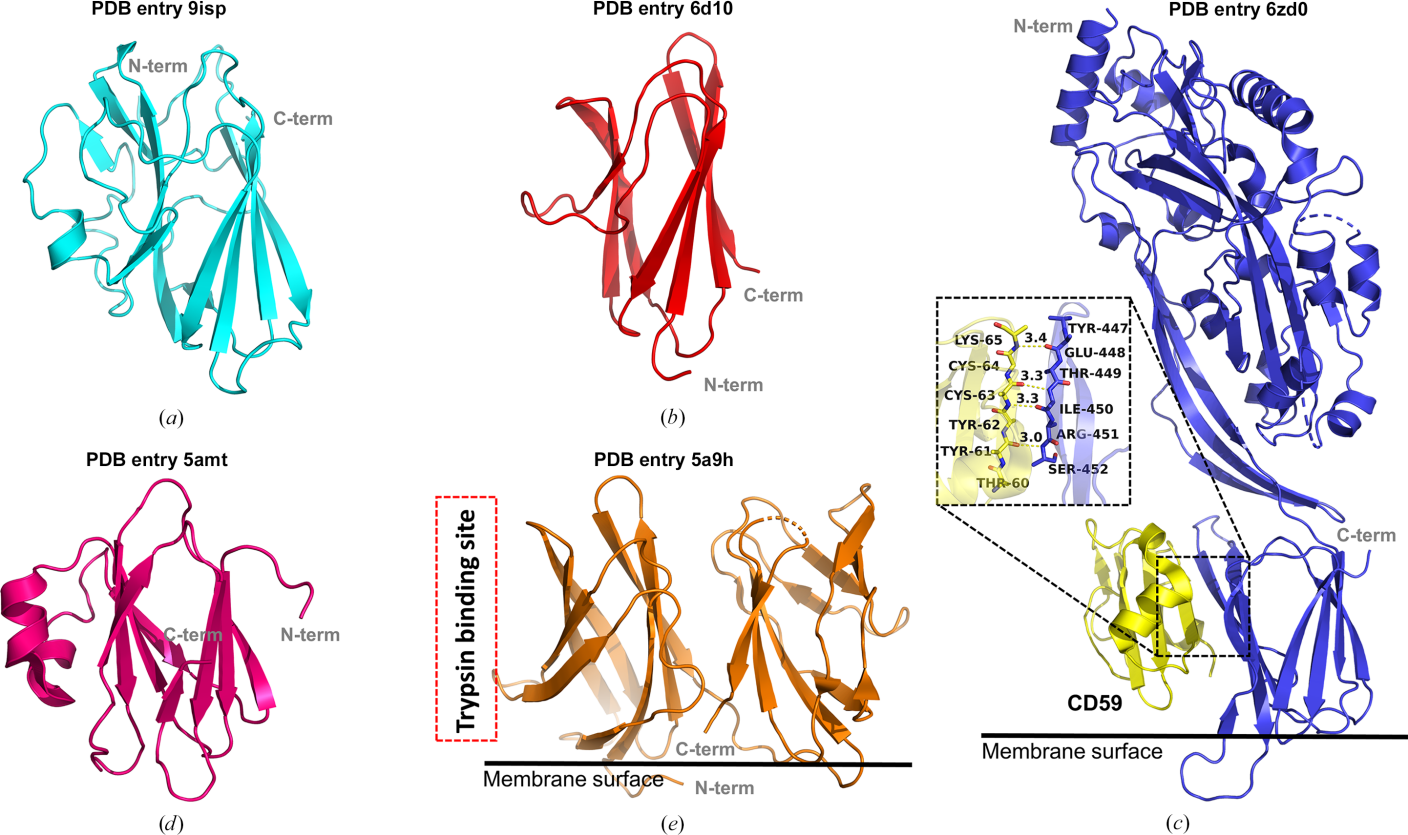
Structure comparison of *Pg*DUF2436 and other structurally similar proteins. (*a*) The structure of *Pg*DUF2436 is presented in cyan. (*b*) The C-terminal domain structure of AlgF (PDB entry 6d10) is colored red. (*c*) The structure of cholesterol-dependent cytolysin from *S. intermedius* (PDB entry 6zd0) is represented in blue. The D4 domain of cholesterol-dependent cytolysin interacts with CD59 (yellow) and the interface is indicated by a red dotted box. (*d*) The structure of IglE (PDB entry 5amt) is presented in hot pink. IglE interacts with β-tubulin, but the interaction site is not yet known. (*e*) The extracellular domain structure of PepT2 (PDB entry 5a9h) is presented in orange. The trypsin binding site of PepT2 is marked with a red dashed box. The black line represents the membrane surface in (*c*) and (*e*).

Structural comparison between *Pg*DUF2436 and other structurally similar proteins such as PDB entries 6d10 (Low *et al.*, 2023 ▸), 5amt (Robb *et al.*, 2010 ▸), 5a9h (Beale *et al.*, 2015 ▸) and 6zd0 (Shah *et al.*, 2020 ▸) (Table 4 ▸) was represented as a cartoon model using *PyMOL* (Fig. 5 ▸; DeLano, 2002 ▸). These proteins share a common structural feature of two antiparallel β-sheets. Investigation of the known functions and binding partners of proteins that are structurally similar to the *Pg*DUF2436 domain revealed that each protein has highly diverse interaction sites and residues involved in binding. However, all of these proteins act as binding modules. Therefore, although the binding partners of the *Pg*DUF2436 domain are not yet known, it is also expected to function as a binding module. In Fig. 5 ▸(*b*), the C-terminal domain of AlgF from *P. aeruginosa* (PDB entry 6d10) is involved in alginate acetylation by binding to the AlgK and AlgX proteins (Low *et al.*, 2023 ▸). In Fig. 5 ▸(*c*), the D4 domain of the cholesterol-dependent cytolysin from *Streptococcus intermedius* (PDB entry 6zd0) interacts through Tyr447, Glu448, Thr449, Ile450, Arg451 and Ser452 with Tyr60, Tyr61, Tyr62, Cys63, Cys64 and Lys65 of human CD59 (glycoprotein; Shah *et al.*, 2020 ▸). Additionally, PDB entry 6zd0 has a membrane-binding site. In Fig. 5 ▸(*e*), the extracellular domain of PepT2 (PDB entry 5a9h) binds to the membrane surface and interacts with trypsin. The red dashed box indicates the trypsin binding site of PepT2 that assists in its peptide-transporter function (Beale *et al.*, 2015 ▸).

**Figure 6:**
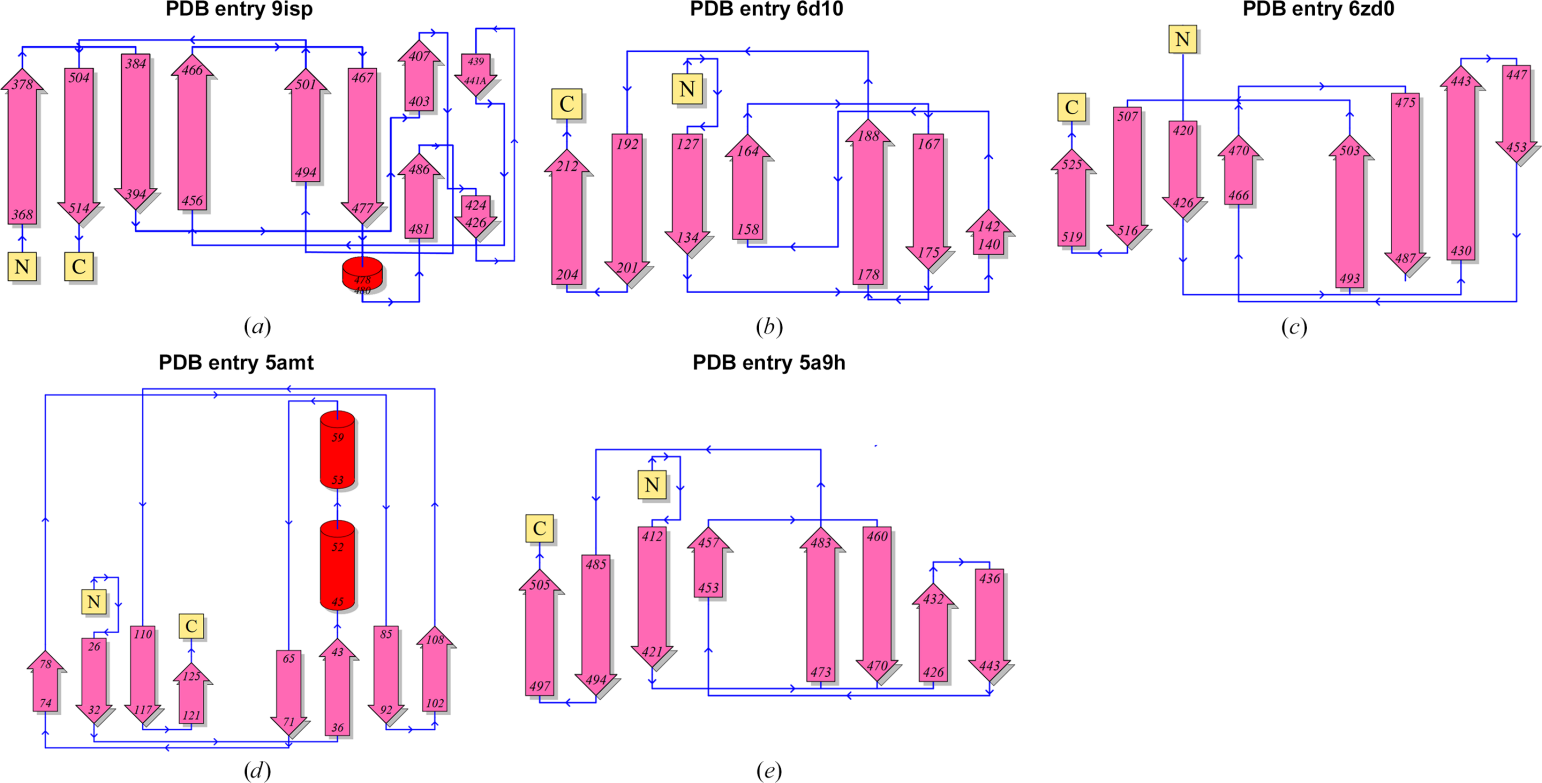
Topology diagrams showing structural differences between *Pg*DUF2436 and the top four proteins from the *DaliLite* server search. Topology diagrams are shown of (*a*) *Pg*DUF2436 (PDB entry 9isp), (*b*) the C-terminal domain structure of AlgF (PDB entry 6d10), (*c*) the D4 domain structure of cholesterol-dependent cytolysin (PDB entry 6zd0), (*d*) IglE (PDB entry 5amt) and (*e*) the extracellular domain structure of PepT2 (PDB entry 5a9h). All diagrams were generated using the *PDBsum* webserver (Laskowski *et al.*, 2018 ▸). In each diagram, ‘N’ represents the N-terminus and ‘C’ represents the C-terminus. Pink arrows indicate β-strands and red circles represent α-helices. Blue lines and arrows indicate the direction of the backbone. The structures of the *Pg*DUF2436 domain and the IglE protein are the only structures with short α-helices, while the other proteins are primarily composed of β-strands.

All of these proteins have the same β-jelly-roll sandwich fold topology, but there is a clear difference in the detailed structure. First of all, the lengths and configurations of each β-strand are different. In addition, *Pg*DUF2436 (PDB entry 9isp) and IglE (PDB entry 5amt; Robb *et al.*, 2010 ▸) have one or two additional short α-helices. The variations in β-strand arrangement and direction among the compared proteins are presented in the topology diagram (Fig. 6 ▸). These distinctions highlight a novel structure for *Pg*DUF2436 with a modified β-jelly-roll sandwich fold.

## 4. Conclusions

In conclusion, this study has elucidated the structure of the *Pg*DUF2436 domain for the first time and comparative studies with other similar structures have been conducted. To thoroughly understand the structure–function relationship of *Pg*DUF2436, we intend to conduct a GST pull-down assay using immobilized fusion-tagged *Pg*DUF2436 as bait to capture potential binding partners. Functional characterization of the DUF2436 domain is expected to provide useful insights for elucidating the pathogenic mechanisms of *P. gingivalis* and developing new drugs against oral diseases.

## 5. Related literature

The following reference is cited in the supporting information for this article: Battye *et al.* (2011 ▸).

## Supplementary Material

PDB reference: *Pg*DUF2436, 9isp

Supplementary Table and Figures. DOI: 10.1107/S2053230X24008185/yg5009sup1.pdf

## Figures and Tables

**Table 1:** Recombinant *Pg*DUF2436 protein-production information

Source organism	*Porphyromonas gingivalis* W83
DNA source	Gene synthesis and cloning
Forward primer	DUF2436-Forward (AGCAGCCATATGATGAGCCGCG)
Reverse primer	DUF2436-Reverse (AGCAGCCTCGAGTTAACACACC)
Cloning vector name	pET-28a
Expression host	*Escherichia coli* BL21 (DE3)
5′ enzyme	NdeI
3′ enzyme	XhoI
Complete DNA sequence	ATGAGCCGCGAAGTTAAACGGATTGGAGATGGTCTGTTCGTAACGATAGAACCTGCAAACGATGTACGTGCAAATGAGGCGAAAGTCGTCTTGGCAGCAGATAACGTGTGGGGTGATAATACCGGTTACCAGTTCCTTCTGGACGCTGACCACAACACCTTCGGCTCTGTTATCCCCGCCACTGGACCTTTATTTACAGGGACCGCAAGCAGTGACCTGTACAGCGCTAATTTTGAATACTTAATACCGGCAAATGCGGATCCGGTTGTGACGACACAGAATATCATTGTGACCGGGCAAGGTGAGGTCGTAATTCCAGGTGGTGTGTATGACTATTGTATCACCAACCCAGAACCGGCATCCGGGAAAATGTGGATTGCAGGAGATGGCGGTAATCAACCGGCCAGATATGACGATTTTACATTTGAAGCAGGTAAAAAATATACTTTTACTATGCGCCGTGCTGGCATGGGGGATGGCACAGATATGGAGGTTGAAGATGATTCACCAGCAAGTTATACGTACACCGTTTATCGTGATGGTACGAAGATTAAAGAAGGACTGACGGAGACTACATATCGTGATGCCGGTCTCTCTGCTCAGTCGCATGAATATTGCGTAGAAGTTAAATATACTGCGGGAGTTTCACCTAAGGTGTGTTAA
Protein sequence	MSREVKRIGDGLFVTIEPANDVRANEAKVVLAADNVWGDNTGYQFLLDADHNTFGSVIPATGPLFTGTASSDLYSANFEYLIPANADPVVTTQNIIVTGQGEVVIPGGVYDYCITNPEPASGKMWIAGDGGNQPARYDDFTFEAGKKYTFTMRRAGMGDGTDMEVEDDSPASYTYTVYRDGTKIKEGLTETTYRDAGLSAQSHEYCVEVKYTAGVSPKVC

**Table 2:** Crystallization details

Method	Vapor diffusion
Plate type	MRC 2-lens crystallization plate (sitting drop)
Temperature (K)	293
Crystallization protein concentration (mg ml^−1^)	90
Storage-buffer composition	20 m*M* Tris–HCl pH 8.0, 200 m*M* NaCl
Mother-liquor composition	0.2 *M* sodium chloride, 0.1 *M* Tris–HCl pH 7, 1 *M* sodium citrate
Drop volume (nl)	600
Reservoir volume (µl)	80

**Table 3:** Data-collection and refinement statistics for *Pg*DUF2436 Values in parentheses are for the outer shell.

Data collection	
X-ray source	7A-SB I, PAL
Space group	*P*3_2_21
*a*, *b*, *c* (Å)	101.28, 101.28, 70.17
α, β, γ (°)	90, 90, 120
Wavelength (Å)	0.979
Resolution (Å)	29.24–2.21 (2.29–2.21)
Total reflections	418427 (36720)
Unique reflections	21119 (2066)
Average *I*/σ(*I*)	22.29 (1.57)
*R*_merge_†	0.39 (2.51)
Multiplicity	19.8
Completeness (%)	99.89 (99.90)
Refinement	
Resolution range (Å)	29.24–2.21 (2.29–2.21)
No. of reflections in working set	21119 (2066)
No. of reflections in test set	1020 (106)
No. of amino-acid residues	156
No. of water molecules	91
*R*_cryst_‡	0.2376 (0.2980)
*R*_free_§	0.2585 (0.3205)
R.m.s.d., bond lengths (Å)	0.01
R.m.s.d., bond angles (°)	1.25
Average *B* value, protein (Å^2^)	40.01
Average *B* value, solvent (Å^2^)	44.16

† *R*_merge_ = 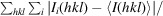

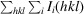
.

‡ *R*_cryst_ = 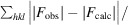

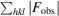
.

§ *R*_free_ was calculated using 5% of all reflections excluded from the refinement stages using high-resolution data.

**Table 4:** Structural homolog search results for *Pg*DUF2436 using the *DaliLite* server

Protein	PDB code	*Dali Z*-score	UniProtKB code	Sequence identity (%) with *Pg*DUF2436 (No. of aligned residues)	Biological function	Reference
C-terminal domain of AlgF from *P. aeruginosa*	6d10	5.9	Q06062	9 (77/89)	Adaptor protein	Low *et al.* (2023 ▸)
Intracellular growth locus E protein (IglE) from *F. tularensis*	5amt	5.4	A0Q7H2	13 (91/105)	Interacts with β-tubulin	Robb *et al.* (2010 ▸)
Extracellular domain of PepT2	5a9h	5.4	Q63424	7 (81/189)	Interacts with trypsin	Beale *et al.* (2015 ▸)
Domain 4 (D4) of cholesterol-dependent cytolysin	6zd0	5.4	Q9LCB8	5 (83/457)	Cholesterol recognition and CD59 binding	Shah *et al.* (2020 ▸)
