# Supplementary material for: First crystal structure of the DUF2436 domain of virulence proteins from Porphyromonas gingivalis
Source: Acta Crystallogr F Struct Biol Commun. 2024 Sep 26;80(Pt 10):252–62. doi: 10.1107/S2053230X24008185 (PMC11448926; doi:10.1107/S2053230X24008185)
Supplement: Supplementary file 1 [file f-80-00252-sup1.pdf]

Acta Cryst F

STRUCTURAL BIOLOGY COMMUNICATIONS

Volume 80 (2024)

Supporting information for article:

First crystal structure of the DUF2436 domain in virulence proteins from *Porphyromonas gingivalis*

Bogeun Kim, Jisub Hwang, Sehyeok Im, Hackwon Do, Youn-Soo Shim and Jun Hyuck Lee

Table S1 Structural analogs in PDB (as identified by TM-align, COFATOR program).

PDB	TM-score	RMSD	Identity	Coverage	Reference
4A1R	0.49	3.63	7.5 %	0.675	(Rao *et al.*, 2011)
3RX9	0.48	3.86	7.1 %	0.688	(Felisberto-Rodrigues *et al.*, 2011)
1JZI	0.45	3.97	8.0 %	0.656	(Crane *et al.*, 2001)

Identity: the percentage of sequence identity in the structurally aligned region.

Coverage: represents the coverage of the alignment by TM-align and is equal to the number of structurally aligned residues divided by length of the query protein (Roy *et al.*, 2012, Zhang *et al.*, 2017).

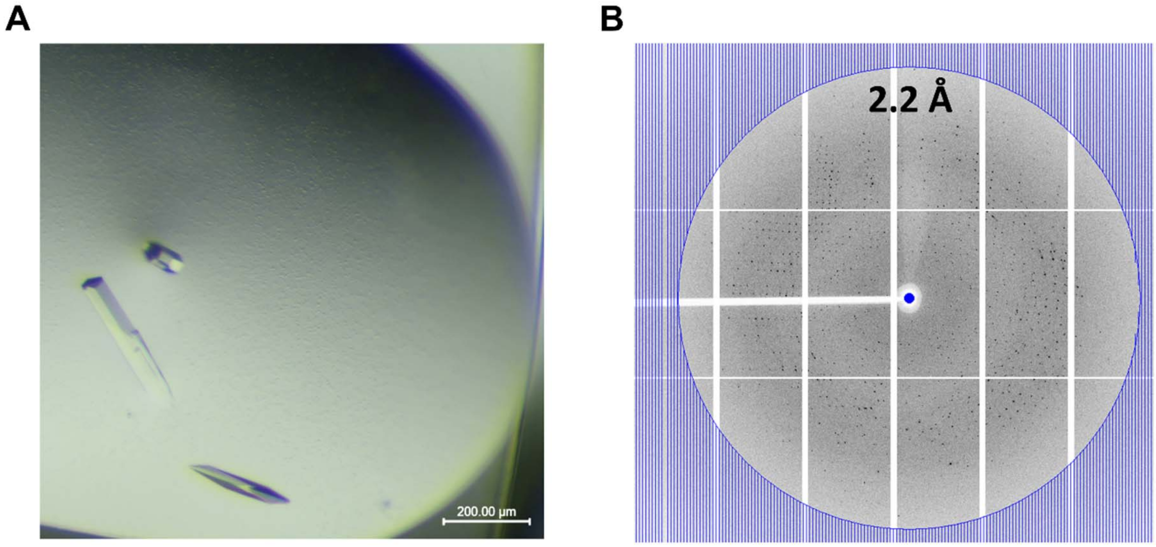

Figure S1 Crystallization and X-ray diffraction pattern of *Pg*DUF2436. (A) Crystals of *Pg*DUF2436 grown after a week using the condition of 0.2 M sodium chloride, 0.1 M Tris-HCl (pH 7.0), and 1 M sodium citrate. (B) Representative X-ray diffraction image of *Pg*DUF2436 crystal with the highest resolution of 2.2 Å (blue circle). The successful diffraction of crystals was tracked and visualized using the iMOSFLM program (Battye *et al.*, 2011).

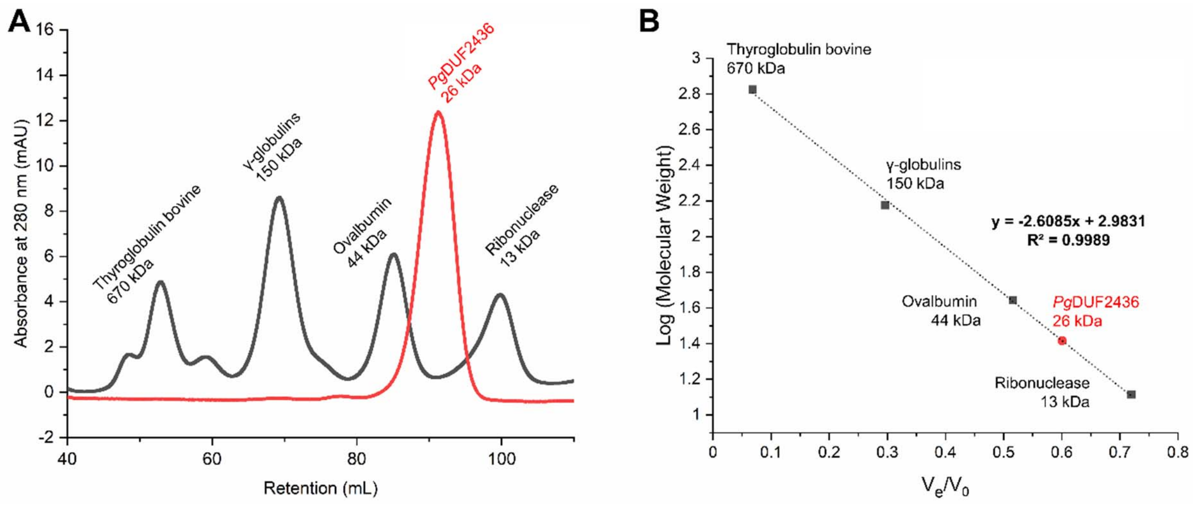

Figure S2 Oligomeric state of *Pg*DUF2436 in solution. (A) Size-exclusion chromatography of protein standard mixture (black) and *Pg*DUF2436 (red). (B) Molecular weight of *Pg*DUF2436 Calculated by linear regression analysis. For gel-filtration chromatography, the protein standard mixture, including Ribonuclease (13 kDa), Ovalbumin (44 kDa), γ-globulins (150 kDa), and Thyroglobulin bovine (670 kDa) was eluted in 20 mM Tris-HCl (pH 8.0) and 200 mM NaCl using HiLoad 16/600 Superdex 200 column and the standard curve was generated.

References

Battye, T. G. G., Kontogiannis, L., Johnson, O., Powell, H. R. & Leslie, A. G. W. (2011). *Acta Crystallographica Section D-Biological Crystallography* **67**, 271-281.

Crane, B. R., Di Bilio, A. J., Winkler, J. R. & Gray, H. B. (2001). *Journal of the American Chemical Society* **123**, 11623-11631.

Felisberto-Rodrigues, C., Durand, E., Aschtgen, M.-S., Blangy, S., Ortiz-Lombardia, M., Douzi, B., Cambillau, C. & Cascales, E. (2011). *PLoS pathogens* **7**, e1002386.

Rao, V. A., Shepherd, S. M., English, G., Coulthurst, S. J. & Hunter, W. N. (2011). *Acta Crystallographica Section D: Biological Crystallography* **67**, 1065-1072.

Roy, A., Yang, J. & Zhang, Y. (2012). *Nucleic Acids Res* **40**, W471-W477.

Zhang, C., Freddolino, P. L. & Zhang, Y. (2017). *Nucleic Acids Res* **45**, W291-W299.
